# Changes in cognitive function in patients with intractable dizziness following vestibular rehabilitation

**DOI:** 10.1038/s41598-018-28350-9

**Published:** 2018-07-03

**Authors:** Nagisa Sugaya, Miki Arai, Fumiyuki Goto

**Affiliations:** 1 Unit of Public Health and Preventive Medicine, School of Medicine, Yokohama City University, Yokohama, Japan; 2 Department of Otolaryngology, National Hospital Organization Tokyo Medical Center, Tokyo, Japan

## Abstract

The purpose of the present study was to investigate changes in cognitive functions, including visuospatial ability, attention, and executive function in patients with intractable dizziness following vestibular rehabilitation. The correlations between improvements in cognitive function and dizziness-related variables and emotional distress were also explored. During hospitalization for 5 days, participants were trained on a vestibular rehabilitation program. Participants completed questionnaires including the Dizziness Handicap Inventory (DHI), Hospital Anxiety and Depression Scale (HADS), and Trail Making Test (TMT), which were used to assess cognitive function. The center of gravity fluctuation measurement and timed up and go test (TUG), which were objective dizziness severity indexes, were performed before, 1 month after, and 4 months after hospitalization. Following vestibular rehabilitation, participants exhibited a significant improvement in the TMT, DHI, HADS, and TUG scores. Correlation analysis between the variables at each time point indicated that TMT scores positively correlated with TUG at baseline. The correlation between changes observed in the TUG and TMT scores was not significant. The degree of improvement of the TUG score did not bear a linear relationship with that of the TMT scores. However, these correlation results were not completely consistent with those in the multiply imputed dataset.

## Introduction

Previous studies have reported an association between vestibular dysfunction and various forms of cognitive impairments, including visuospatial ability, attention, executive function, and memory^1,2^. Visuospatial cognition is the cognitive domain that is most often studied in human vestibular research^3–5^. Although the mechanism of the association between vestibular dysfunction and cognitive impairments is still unclear, several potential pathways have been hypothesized^1^. For example, vestibular dysfunction may lead to atrophy of areas of the cortical vestibular network, including the hippocampus, which may in turn be responsible for the deterioration of memory and visuospatial ability^6–8^.

Currently, there is a widespread consensus that exercise-based therapy known as ‘vestibular rehabilitation’ is the most effective treatment for dizziness linked to vestibular dysfunction^9^. We have previously reported that vestibular rehabilitation contributes to the improvement of perceived handicap due to dizziness and psychological distress^10^. Given the relationship between the vestibular system and cognitive function, it is possible to speculate that improvement in dizziness by vestibular rehabilitation may ameliorate cognitive function. Additionally, the high prevalence of affective disorders in individuals with vestibular impairment may also contribute to cognitive dysfunction^11^. Thus, both improvements in dizziness and emotional distress by vestibular rehabilitation may contribute to changes in cognitive function in patients with intractable dizziness.

The purpose of the present study was to investigate changes in cognitive functions, including visuospatial ability, attention, and executive function in patients with intractable dizziness following vestibular rehabilitation, and the correlation between improvements of cognitive function, dizziness-related variables, and emotional distress.

## Methods

### Participants

The present study recruited patients with a chief complaint of dizziness who visited the Department of Otorhinolaryngology at the National Tokyo Medical Center between February 2015 and September 2016. Participants reported that they felt persistent dizziness even after conventional treatment in Japan, which included the following: (1) drug therapy with 36 mg betahistine^12^ daily for the first 2–4 weeks, (2) lifestyle counseling to exercise daily, including walking, and (3) sleeping sufficiently and reducing stress. We recruited participants for the present study from this pool of patients if they met the following criteria: (1) patient was ≥20 years old; (2) dizziness had persisted for at least 3 months despite conventional treatment mentioned above in the outpatient clinic; (3) the patient wished to have intensive, inpatient therapy for persistent dizziness; (4) the patient had not experienced vestibular rehabilitation before the starting the intervention; and (5) the patient was literate. Our exclusion criteria were as follows: (1) a diagnosis of dizziness due to cerebrovascular disorder; (2) medical contraindications for making the necessary head movements during vestibular rehabilitation (e.g., severe cervical disorder); (3) serious comorbidity (e.g., a life-threatening condition, severe cognitive impairment, or severe psychiatric disorder); (4) central nervous system disease; or (5) bilateral vestibular deficit.

Patients underwent pure tone audiometry, vestibular investigation (including eye movements), posturography, head impulse test, video head impulse test, electronystagmography, auditory brainstem response, computed tomography, and/or magnetic resonance imaging as necessary for the diagnosis. The clinical diagnosis was defined based on the results of these examinations. Canal dysfunction was confirmed in patients with vestibular neuritis and unilateral vestibulopathy.

The present study was approved by the ethical committee of the National Tokyo Medical Center (R12–009) and has been performed in accordance with the ethical standards laid down in the 1964 Declaration of Helsinki and its later amendments.

### Measures

#### Trail Making Test

The Trail Making Test (TMT) is used to assess visuospatial scanning, attention, processing speed, and executive function. In the TMT-A, participants were asked to connect a series of numbers in consecutive order (1, 2, 3, etc.). The TMT-A examines visual scanning ability, attention, and processing speed. In the TMT-B, participants were required to connect a series of letters and numbers in alternating consecutive order (1, A, 2, B, 3, C, etc.). The TMT B examines executive function, visual scanning ability, attention, and processing speed. The time in seconds to complete the task was recorded^13^. We calculated the difference by subtracting the TMT-A score from the TMT-B score (TMT-B-A)^14^. The TMT-B-A score was reportedly minimizes visuo-perceptual and working memory demands, providing a relatively pure indicator of executive control abilities^15^.

#### Dizziness Handicap Inventory

The Dizziness Handicap Inventory^16,17^ is a standard questionnaire that quantitatively evaluates the degree of handicap in the daily lives of patients with vestibular disorders; it consists of 25 questions. The total score ranges from 0 (no disability) to 100 (severe disability).

#### The center of gravity fluctuation measure

The center of gravity fluctuation measure for objective assessment of the severity of dizziness was performed using a stabilometer (G-5000, Anima Corp., Tokyo); it provided the total path length (LNG) and environmental area (ENV) during quiet stance with eyes open and eyes closed for 60 s (the LNG is same as the velocity of sway path value multiplied by 60 s).

#### Timed up and go test

The timed up and go test (TUG test)^18^ assesses functional mobility consisting of basic motor agility and dynamic balance. During the test, patients were required to stand up from a chair, walk 3 m, turn, walk back, and sit down. We used a chair without armrests since obese patients found it difficult to sit against armrests. Participants freely selected the direction of the turn by themselves. The time needed to perform this task was recorded twice. The smaller value was registered as the TUG test score.

#### Hospital Anxiety and Depression Scale

The Hospital Anxiety and Depression Scale (HADS)^19,20^ is a self-reported questionnaire containing 14 questions scored on a 4-point scale, consisting of an anxiety subscale and depression subscale with seven items each. This psychometric instrument was chosen because all its items refer solely to an emotional state and do not consider somatic symptoms.

### The intervention

Patients were hospitalized for 5 days in groups of 8–10 individuals. During this time, the groups were trained to perform the 30-min vestibular rehabilitation program by themselves^21^. The program consisted of head and eye exercises in a sitting or standing position. Exercises in the sitting position included the following seven exercises: (1) quick horizontal eye movement; (2) quick vertical eye movement; (3) eye tracking horizontal direction; (4) eye tracking vertical direction; (5) horizontal head shaking with gazing fixed target; (6) vertical head shaking with gazing fixed target; and (7) oblique head tilting with gazing fixed target. Each eye or head movement was repeated 20 times per session. Exercise in a standing position consisted of the following 13 exercises: (1) standing up and sitting down with eyes open, three times; (2) standing up and sitting down with eyes closed, three times; (3) standing with eyes closed and feet apart for 20 s; (4) standing with eyes closed and feet together for 20 s; (5) in tandem stance with right foot in front for 20 s; (6) in tandem stance with left foot in front for 20 s; (7) one leg stand on the right foot for 20 s; (8) one leg stand on the left foot for 20 s; (9) 180° turn to the left, three times; (10) 180° turn to the right, 3 times; (11) walking 10 m with tandem gait; (12) walking 10 m with horizontal head shakes; and (13) walking 10 m with vertical head shakes. During training, patients performed these exercises three times a day under the supervision of a physician. After 5 days, all patients had learned how to perform the exercises. The patients were then instructed to continue performing the vestibular rehabilitation program three times a day after discharge. All participants were asked to record their exercises after discharge from hospital, and physicians verbally confirmed participant progress at every visit.

### Procedure

After the participants had provided written, informed consent, they were evaluated on the day of hospitalization (time 1), as well as at 1 month and 4 months after hospitalization (time 2 and time 3, respectively), using the above-mentioned questionnaires. The TMT, static posturography, and TUG were also conducted. All drugs that could affect dizziness, including vestibular suppressants, were terminated soon after the introduction of vestibular rehabilitation.

### Statistical analyses

Data analyses were performed using the SPSS 22.0 software (SPSS, Chicago, IL, USA). The primary analysis consisted of repeated measures analysis of variance (ANOVA) to analyze the effects of time on all outcomes, and correlation analysis (Pearson’s correlation coefficient) to examine the relationship between scores at each time point and changes in outcomes during rehabilitation. Additionally, correlation analysis between each score at time 1, and changes in scores from time 1 to time 3, were performed as the secondary analysis based on the results obtained. The significance level for the ANOVA was set at less than 5%. Multiple testing corrections for correlation analyses were performed using the Bonferroni test. The significance level for correlation between scores at each time point was *p* < 0.004 (=0.05/12 [3 time points * 3 sub-scores of the TMT and age]) after the correction. The significance level for correlation between changes in outcomes during rehabilitation was *p* < 0.016 (=0.05/3 sub-scores of the TMT) after correction. The significance level for correlation between each score at time 1 and changes in the scores from time 1 to time 3, and partial correlation was *p* < 0.004 (=0.05/11 variables) after the correction.

Additionally, we applied an intention-to-treat (ITT) analysis using a multiple imputation technique^22^ to create and analyze multiply imputed datasets. Data were missing for 71 of the 131 participants. The incomplete variables were as follows: (1) 7 center of gravity fluctuation measures and 2 TUG results at time 1, (2) 1 center of gravity fluctuation measure and 7 TUG results at time 2, and (3) 46 TMT results, 34 DHI scores, 33 center of gravity fluctuation measures, 40 TUG results, and 34 HADS scores at time 3.

Multiple imputation was estimated using Bayesian linear regression. We averaged and combined the 20 imputed datasets. We conducted primary tests, including the repeated measures ANOVA, to analyze the difference in the outcomes among all time points. We performed a correlation analysis to examine the relationship among the outcomes at each time point and among the changes in these outcomes from time 1 to time 3 using multiply imputed datasets.

### Data availability

The datasets generated during and/or analyzed during the current study are available from the corresponding author on reasonable request.

## Results

### Participant characteristics

**Figure 1:**
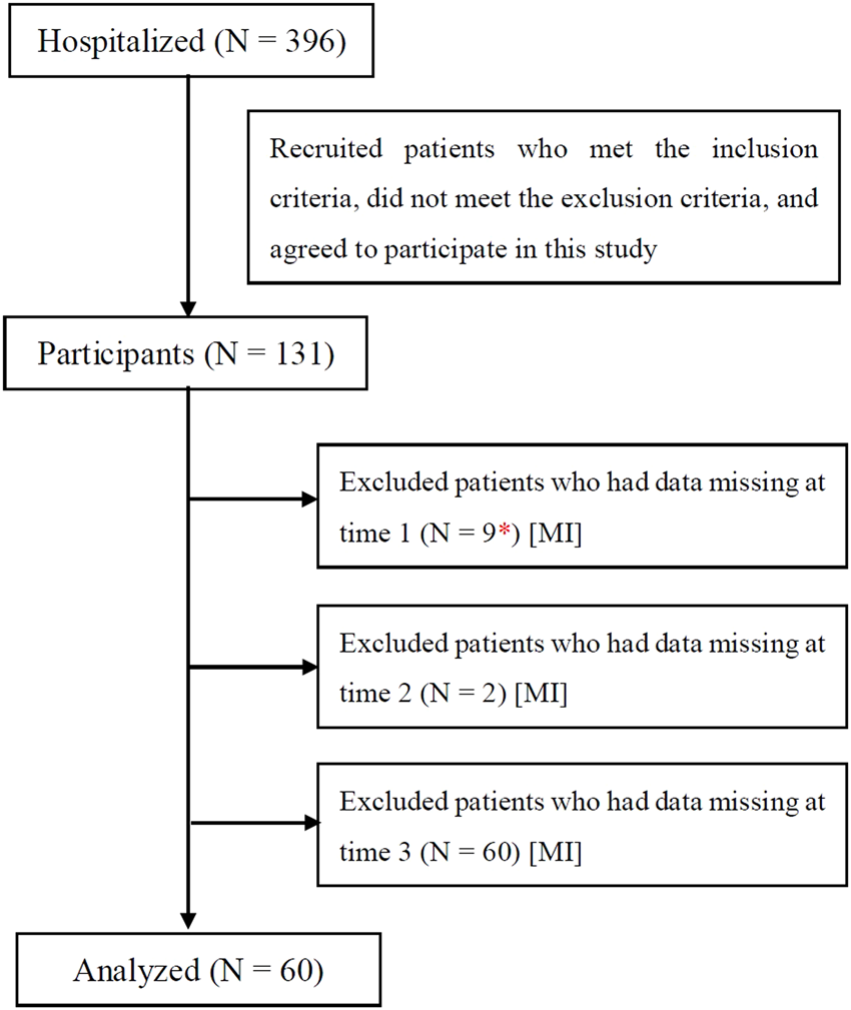
Flow chart of participants in the present study. [MI]: participants applied to multiple imputation analyses. *Data were missing for 5 of the 9 participants at time 2, whereas data were missing for 3 of the 9 participants at time 3.

During the study period, 396 patients with dizziness were hospitalized, of which 131 patients (32 male and 99 female patients) met the inclusion criteria and agreed to participate in the present study. We further excluded those who had data missing at any time of the examination (n = 71, 22 male and 49 female patients); thus, 60 patients (10 male and 50 female patients, mean age = 55.9 ± 15.3 years) were included in the final analysis (Fig. 1). Table 1 outlines the diagnoses of the participants, according to a medical history recorded during their initial visit.

**Table 1:** Participant diagnoses.

Diagnosis	Total
Vestibular neuritis	11
Unilateral vestibulopathy	8
BPPV (HC or PC)	8
Meniere’s disease	8
Sudden deafness with vertigo	7
Vestibular migraine	5
Psychogenic dizziness	4
Post-traumatic dizziness	3
Recurrent vestibulopathy	2
Other	4
Total	60

BPPV, benign paroxysmal positional vertigo; HC, horizontal canal type; PC, posterior canal type.

### Change of each variable by vestibular rehabilitation

Table 2 summarizes the results of the repeated measures ANOVA.

**Table 2:** Comparison of each variable between time points in 60 participants.

	Time 1	Time 2	Time 3	*F*	*p*
	Mean ± SD	Mean ± SD	Mean ± SD		
TMT-A	35.3 ± 14.9	32.2 ± 12.1	28.4 ± 12.4	13.4	<0.0001
TMT-B	85.2 ± 36.4	64.2 ± 22.6	63.9 ± 25.0	38.9	<0.0001
TMT-B-A	49.9 ± 27.2	32.0 ± 16.6	35.5 ± 18.6	25.8	<0.0001
DHI	47.3 ± 21.0	27.2 ± 19.8	23.1 ± 20.3	76.3	<0.0001
LNG during eye-opening	96.6 ± 41.7	91.6 ± 33.7	88.5 ± 32.1	2.9	0.07
LNG during eye-closing	132.5 ± 84.7	123.4 ± 63.1	112.3 ± 49.1	4.9	0.01
ENV during eye-opening	4.6 ± 3.9	4.7 ± 4.2	4.2 ± 3.3	0.7	0.44
ENV during eye-closing	7.1 ± 9.3	6.0 ± 8.3	5.0 ± 4.3	3.0	0.07
TUG	6.9 ± 1.1	5.9 ± 1.0	6.0 ± 1.2	62.5	<0.0001
HADS-A	7.8 ± 5.7	5.0 ± 3.9	5.2 ± 4.4	20.5	<0.0001
HADS-D	7.1 ± 4.0	5.2 ± 3.6	5.0 ± 3.6	15.1	<0.0001

DHI, Dizziness Handicap Inventory; ENV, environmental area; HADS-A, Hospital Anxiety and Depression Scale-Anxiety; HADS-D, Hospital Anxiety and Depression Scale-Depression; LNG, total length of path; SD, standard deviation; TMT, Trail Making Test; during TMT-A participants were asked to connect a series of numbers in consecutive order (1, 2, 3, etc.); during TMT-B participants were required to connect a series of letters and numbers in an alternating consecutive order (1, A, 2, B, 3, C, etc.); TMT-A-B is the score difference between TMT-A and TMT-B; TUG, timed up and go test.

Results of the post-hoc test: TMT-A: Time 1 & 2 > 3.

TMT-B, TUG, and HADS: Time 1 > 2 & 3.

DHI: Time 1 > 2 > 3.

LNG during eye-closing: Time 1 > 3.

Regarding the TMT scores, there was a significant main effect of time in the TMT-A, TMT-B, and TMT-B-A scores. The post-hoc test showed that the TMT-A score at time 3 was significantly lower than that at time 1 (*p* < 0.0001) and time 2 (*p* = 0.03), while the TMT-B and TMT-B-A scores at time 2 (*p* < 0.0001) and time 3 (*p* < 0.0001) were significantly lower than at time 1.

Furthermore, a significant main effect of time on the DHI score was found, and the post-hoc test revealed that the score at time 3 was significantly lower than that at time 1 (*p* < 0.0001) and time 2 (*p* = 0.04). Further, the score at time 2 was also significantly lower than that at time 1 (*p* < 0.0001).

Regarding the center of gravity fluctuation measure, there was a significant main effect of time in the LNG during eye-closing; the post-hoc test revealed a significantly lower score at time 3 than at time 1 (*p* = 0.03). No significant differences in other LNG and ENV variables were found.

A significant main effect of time on the TUG test score was also found and the post-hoc analysis revealed significantly lower scores at time 2 (*p* < 0.0001) and time 3 (*p* < 0.0001) than at time 1.

In terms of the HADS, significant main effects of time in both the HADS-anxiety (HADS-A) and HADS-depression (HADS-D) scores were observed. The post-hoc analysis revealed significantly lower scores at time 2 (HADS-A: *p* < 0.0001; HADS-D: *p* = 0.0005) and time 3 (HADS-A: *p* < 0.0001; HADS-D: *p* < 0.0001) than at time 1.

The ITT analysis of multiply imputed datasets (N = 131) showed significant main effects of time in all sub-scores of the TMT (TMT-A: *F* = 11.89, *p* < 0.0001, TMT-B: *F* = 27.90, *p* < 0.0001, TMT-B-A: *F* = 25.82, *p < *0.0001), DHI (*F* = 111.84, *p* < 0.0001), TUG (*F* = 94.86, *p* < 0.0001), and two sub-scores of the HADS (HADS-A: *F* = 30.11, *p* < 0.0001, HADS-D: *F* = 21.60, *p* < 0.0001). There were no significant differences in the LNG and ENV variables. Regarding the DHI, the post-hoc analysis revealed that the scores at time 2 (*p* < 0.0001) and 3 (*p* < 0.0001) were significantly lower than those at time 1, and that the score at time 3 was significantly lower than that at time 2 (*p* = 0.03). Regarding the post-hoc analyses in other variables, the scores at time 2 (TMT-A: p = 0.005, other: *p* < 0.0001) and 3 (*p* < 0.0001) were significantly lower than those at time 1.

### Correlation between the variables at each time point

Table 3 summarizes the results of the correlation analysis between the variables at each time-point. Age significantly correlated with the TMT-A and B scores at time 1 and time 2, and the TMT-B and B-A scores at time 3. The TUG score significantly correlated with the TMT-A, B, and B-A scores at time 1.

**Table 3:** Correlation between variables at each time point in 60 participants.

		Age	DHI	LNG during eye-opening	LNG during eye-closing	ENV during eye-opening	ENV during eye-closing	TUG	HADS-A	HADS-D
*Time 1*	TMT-A	0.41*	−0.14	0.07	−0.03	−0.12	−0.16	0.37*	0.01	0.01
	TMT-B	0.43*	0.04	0.03	0.00	−0.20	−0.18	0.52*	0.08	0.06
	TMT-B-A	0.35	0.12	0.00	0.02	−0.21	−0.15	0.50*	0.10	0.08
	Age	—	−0.20	0.12	0.09	−0.18	−0.21	0.35	−0.34	−0.20
*Time 2*	TMT-A	0.45*	−0.01	0.03	−0.05	−0.21	−0.25	0.20	0.22	0.28
	TMT-B	0.43*	0.07	0.09	0.02	−0.20	−0.22	0.27	0.21	0.26
	TMT-B-A	0.27	0.11	0.10	0.06	−0.11	−0.12	0.23	0.13	0.14
	Age	—	−0.11	0.20	0.12	−0.22	−0.27	0.17	−0.16	0.02
*Time 3*	TMT-A	0.30	0.10	0.02	0.00	−0.09	−0.13	0.26	0.15	0.29
	TMT-B	0.45*	0.05	0.03	−0.04	−0.10	−0.17	0.29	0.14	0.18
	TMT-B-A	0.40*	0.00	0.02	−0.05	−0.07	−0.14	0.22	0.09	0.05
	Age	—	−0.12	0.18	0.06	−0.15	−0.22	0.25	−0.22	−0.12

*p < 0.004 (after Bonferroni’s adjustment).

DHI, Dizziness Handicap Inventory; ENV, environmental area; HADS-A, Hospital Anxiety and Depression Scale-Anxiety; HADS-D, Hospital Anxiety and Depression Scale-Depression; LNG, total length of path; SD, standard deviation; TMT, Trail Making Test; during TMT-A participants were asked to connect a series of numbers in consecutive order (1, 2, 3, etc.); during TMT-B participants were required to connect a series of letters and numbers in alternating consecutive order (1, A, 2, B, 3, C, etc.); TMT-A-B is the score difference between TMT-A and TMT-B; TUG, timed up and go test.

The ITT analysis of multiply imputed datasets (N = 131) showed that the TUG score significantly correlated with the TMT-A score at time 3 (*r* = 0.39), TMT-B scores at times 1 (*r* = 0.29) and 3 (*r* = 0.34), and TMT-B-A score at time 1 (*r* = 0.50). The TMT-A score significantly correlated with LNG during eye-closing at time 3 (*r* = 0.30). Age significantly correlated with the TMT-A and B at time 1 (A: *r* = 0.37; B: *r* = 0.31), 2 (A: *r* = 0.33; B: *r* = 0.37), and 3 (A: *r* = 0.42; B: *r* = 0.46), the TMT-B-A at time 2 (*r* = 0.26) and 3 (*r* = 0.35), LNG during eye-opening at time 2 (*r* = 0.27), the TUG scores at time 1 (*r* = 0.38) and 3 (*r* = 0.33), and HADS scores at times 1 (*r* = −0.25), 2 (*r* = −0.25), and 3 (*r* = −0.32). The significant correlation between the TMT-A score and TUG score at time 1 found in 60 participants disappeared in the multiply imputed dataset.

### Correlation between time-point changes in the TMT scores and other variables

Table 4 summarizes the results of the correlation analysis between changes from time 1 to time 3 in the TMT scores and those in other variables. The changes of the TMT-A score significantly and negatively correlated with those of the DHI, HADS-A, and HADS-D scores. The changes of the TMT-B-A score did not significantly correlate with changes of any other variables.

**Table 4:** Correlation between changes from time 1 to time 3 in the TMT scores and those in other variables in 60 participants.

	DHI	LNG during eye-opening	LNG during eye-closing	ENV during eye-opening	ENV during eye-closing	TUG	HADS-A	HADS-D
TMT-A	−0.36*	−0.13	−0.10	−0.11	−0.06	0.19	−0.38*	−0.38*
TMT-B	−0.19	−0.20	−0.06	−0.26	−0.09	0.20	−0.08	−0.11
TMT-B-A	−0.07	−0.17	−0.02	−0.24	−0.07	0.14	0.07	0.04

*p < 0.016 (after Bonferroni’s adjustment).

DHI, Dizziness Handicap Inventory; ENV, environmental area; HADS-A, Hospital Anxiety and Depression Scale-Anxiety; HADS-D, Hospital Anxiety and Depression Scale-Depression; LNG, total length of path; SD, standard deviation; TMT, Trail Making Test; during TMT-A participants were asked to connect a series of numbers in consecutive order (1, 2, 3, etc.); during TMT-B participants were required to connect a series of letters and numbers in alternating consecutive order (1, A, 2, B, 3, C, etc.); TMT-A-B is the score difference between TMT-A and TMT-B; TUG, timed up and go test.

The ITT analysis of multiply imputed datasets (N = 131) showed that the changes in the TMT-B and TMT-B-A significantly correlated with those in LNG during eye-opening (B: *r* = −0.32; B-A: *r* = −0.33), ENV during eye-opening (B: *r* = −0.27; B-A: *r* = −0.24), and ENV during eye-closing (B: *r* = −0.32; B-A: *r* = −0.33), and that the change in the TMT-A significantly correlated with that in the TUG (*r* = −0.24). The significant correlation of the change in the TMT-A score with that in the DHI, HADS-A, and HADS-D found in 60 participants disappeared in multiply imputed dataset.

### Correlation between each score at time 1 and the corresponding changes from time 1 to time 3

Table 3 summarizes the correlation results between each score at time 1 and change in scores from time 1 to time 3. All scores except the TUG at time 1 significantly and positively correlated with their corresponding changes from time 1 to time 3 (see colored area in Table 5). Regarding the relationship between the change of the TMT scores and other variables at baseline (time 1), the change of the TMT-A score significantly and negatively correlated with the HADS-D score. Conversely, changes in the TMT-B and TMT-B-A scores significantly and positively correlated with the TUG scores.

**Table 5:** Correlation between each score at time 1 and changes of those scores from time 1 to time 3 in 60 participants.

	Scores at time 1										
	TMT-A	TMT-B	TMT-B-A	DHI	LNG during eye-opening	LNG during eye-closing	ENV during eye-opening	ENV during eye-closing	TUG	HADS-A	HADS-D
***Changes from time 1 to 3***											
TMT-A	0.55*	0.47*	0.32	−0.34	−0.04	−0.09	−0.07	−0.07	0.12	−0.24	−0.39*
TMT-B	0.36	0.73*	0.79*	−0.02	−0.01	−0.001	−0.16	−0.05	0.40*	0.04	−0.02
TMT-B-A	0.17	0.62*	0.73*	0.12	0.00	0.03	−0.15	−0.03	0.39*	0.14	0.14
DHI	−0.24	−0.10	−0.01	0.47*	0.08	0.12	−0.08	−0.03	−0.22	0.14	0.14
LNG during eye-opening	0.04	−0.09	−0.15	0.13	0.65*	0.52*	0.56*	0.49*	−0.03	−0.06	−0.03
LNG during eye-closing	−0.03	−0.01	0.00	0.10	0.65*	0.82*	0.36	0.44*	−0.02	−0.04	0.003
ENV during eye-opening	−0.08	−0.20	−0.23	−0.04	0.32	0.23	0.65*	0.66*	0.01	−0.12	−0.12
ENV during eye-closing	−0.12	−0.16	−0.15	−0.14	0.37	0.36	0.72*	0.89*	−0.06	−0.12	−0.16
TUG	0.19	0.19	0.15	−0.15	−0.23	−0.30	−0.08	−0.13	0.32	0.16	0.07
HADS-A	−0.15	−0.08	−0.03	0.34	−0.13	−0.12	−0.14	−0.11	0.14	0.64*	0.47*
HADS-D	−0.21	−0.12	−0.05	0.24	−0.15	−0.19	−0.13	−0.06	0.10	0.38*	0.51*

*p < 0.004 (after Bonferroni’s adjustment).

DHI, Dizziness Handicap Inventory; ENV, environmental area; HADS-A, Hospital Anxiety and Depression Scale-Anxiety; HADS-D, Hospital Anxiety and Depression Scale-Depression; LNG, total length of path; SD, standard deviation; TMT, Trail Making Test; during TMT-A participants were asked to connect a series of numbers in consecutive order (1, 2, 3, etc.); during TMT-B participants were required to connect a series of letters and numbers in alternating consecutive order (1, A, 2, B, 3, C, etc.); TMT-A-B is the score difference between TMT-A and TMT-B; TUG, timed up and go test.

## Discussion

In the present study, we demonstrated that patients with intractable dizziness exhibited a significant improvement in cognitive functions including visuospatial ability, attention, and executive function as evidenced by the TMT. These changes also coincided with improvement in dizziness-related indexes and psychological distress following vestibular rehabilitation. Although the mean TMT scores at baseline were relatively higher than previously reported average scores in healthy participants aged 55 and 59 years^23^, those scores became lower than the average scores 3 months after the initiation of vestibular rehabilitation. A previous study reported that balance training improved memory and spatial cognition, but not executive functions, in healthy participants^24^. The presence of affective complications in individuals with vestibular impairments may contribute to cognitive dysfunction^11^. Thus, both improvements of dizziness and emotional distress by vestibular rehabilitation could contribute to changes in cognitive functions including executive function as evidenced by the TMT-B-A and TMT-B. The indexes of the center of gravity fluctuation measure, except for the LNG during eye-closing, were not significantly improved by vestibular rehabilitation. We previously reported that in patients with intractable dizziness, body sway during the eye-open condition^25^ or both during eye-open and eye-close conditions^10^ was not significantly improved by vestibular rehabilitation, which is in contrast to other findings indicating significant improvements of these indexes^26^. Interestingly, participants who exhibited significant improvements of LNG and ENV^26^ had more severe indexes than participants who did not show significant improvements of these indexes, both previously^25^ and in the present study. Thus, LNG and ENV may not be very sensitive to the effect of vestibular rehabilitation on dizziness. In the multiply imputed dataset (N = 131), we found almost the same results as those in the 60 participants. Further, the ANOVA in 60 participants may indicate relatively robust results.

Our results also indicated that visuospatial ability, attention (TMT-A or TMT-B), and executive function (TMT-B-A) positively correlated with functional mobility (TUG) before the initiation of vestibular rehabilitation. Based on these findings, cognitive function, including visuospatial ability, attention, and executive function, could be related to functional mobility, in the presence of prominent dizziness symptoms alone. Further, the correlation could weaken as dizziness symptoms improve. However, the results in the multiply imputed dataset (N = 131) showed significant and positive correlations between the TMT-A and B scores and the TUG score at time 3. Notably, these results were not found in the 60 participants; thus, we should re-examine the correlation results in a larger sample.

However, based on the correlation analysis between changes from baseline to 4 months after the start of vestibular rehabilitation in the TMT scores and those in other variables, the improvement of the TMT-A score was negatively correlated with the improvement of perceived dizziness handicap and emotional distress in the 60 participants. Additionally, more severe states of all variables except the TUG score at baseline were associated with greater corresponding changes between examinations from times 1 to time 3. Furthermore, more severe depression at baseline were associated with smaller changes in the TMT-A scores and decreased perceived dizziness handicap at baseline tend to be related to smaller changes in the TMT scores (*p* = 0.008), while worse functional mobility assessed by the TUG test at baseline was associated with a greater change in the TMT-B and A-B scores. Thus, severe states of emotional difficulties, rather than greater improvement of those variables, could be related to weaker improvement of the visuospatial ability and attention, but not of executive function. However, the significant correlation coefficients reported in the present study were not robust because the results of the correlation analysis for the changes in multiple parameters from time 1 to time 3 observed in the multiply imputed datasets (N = 131) were not consistent with those in the 60 participants. Thus, the results of the correlation analyses should be interpreted with caution.

In addition, following vestibular rehabilitation, patients with intractable dizziness demonstrated a significant improvement in their cognitive functions as evidenced by the TMT scores, with coincident improvement of their functional mobility. In particular, the tendency of executive function improvement (time 1 to times 2 and 3) appeared to be comparable to that of the functional mobility during vestibular rehabilitation. However, although the correlation between improvements in the functional mobility and cognitive functions was positive, this finding was not statistically significant. Thus, the magnitude of improvement in these cognitive function domains could not have correspond with that in functional mobility.

A few limitations in the present study must be noted. First, cognitive functions were evaluated using the TMT alone. Second, we should additionally use other sensitive measures for assessment of functional mobility including the Dynamic Gait Index^27^ and the Functional Gait Assessment^28^ in future research. Third, we did not conduct any assessment during the 5 days in hospital. Fourth, since a low number of the participants were included in the present study we could not analyze our data in consideration of sex and age. Fifth, data for at least one of the outcomes were missing for approximately half of the participants (54.2%), particularly at 4 months after the start of investigation. Sixth, the confirmation of adherence to the home-based program after discharge was not sufficient. Although we asked all participants to record their exercises after discharge and verbally confirmed progress with them at every visit, we did not obtain data of records. Finally, the present study lacked a control group.

## Conclusion

Patients with intractable dizziness demonstrated a significant improvement in cognitive functions including visuospatial ability, attention, and executive function, with coincident improvement of dizziness-related indexes and psychological distress, following vestibular rehabilitation. These cognitive function domains correlated with functional mobility consisting of basic motor agility and dynamic balance before the initiation of vestibular rehabilitation. There was no linear relationship between the degree of improvement of functional mobility and the improvement in cognitive function. However, given the discrepancy between correlation results in the 60 participants and multiply imputed dataset, we should re-examine the correlation results in a larger sample.
